# Mortality predictors of *Staphylococcus aureus* bacteremia: a prospective multicenter study

**DOI:** 10.1186/s12941-016-0122-8

**Published:** 2016-02-09

**Authors:** Mesut Yilmaz, Nazif Elaldi, İlker İnanç Balkan, Ferhat Arslan, Ayşe Alga Batırel, Mustafa Zahir Bakıcı, Mustafa Gokhan Gozel, Sevil Alkan, Aygül Doğan Çelik, Meltem Arzu Yetkin, Hürrem Bodur, Melda Sınırtaş, Halis Akalın, Fatma Aybala Altay, İrfan Şencan, Emel Azak, Sibel Gündeş, Bahadır Ceylan, Recep Öztürk, Hakan Leblebicioglu, Haluk Vahaboglu, Ali Mert

**Affiliations:** Department of Infectious Diseases and Clinical Microbiology, Istanbul Medipol University, TEM Avrupa Otoyolu Göztepe Çıkışı No: 1, Bağcılar, 34214 İstanbul, Turkey; Department of Infectious Diseases and Clinical Microbiology, Faculty of Medicine, Cumhuriyet University, Sivas, Turkey; Department of Medical Microbiology, Faculty of Medicine, Cumhuriyet University, Sivas, Turkey; Department of Infectious Diseases and Clinical Microbiology, Cerrahpaşa Medical Faculty, Istanbul University, Istanbul, Turkey; Department of Infectious Diseases and Clinical Microbiology, Dr. Lütfi Kirdar Kartal Training and Research Hospital, Istanbul, Turkey; Department of Infectious Diseases and Clinical Microbiology, Faculty of Medicine, Trakya University, Edirne, Turkey; Department of Infectious Diseases and Clinical Microbiology, Ankara Numune Training and Research Hospital, Ankara, Turkey; Department of Medical Microbiology, Faculty of Medicine, Uludag University, Bursa, Turkey; Department of Infectious Diseases and Clinical Microbiology, Faculty of Medicine, Uludag University, Bursa, Turkey; Department of Infectious Diseases and Clinical Microbiology, Diskapi Yildirim Beyazit Training and Research Hospital, Ankara, Turkey; Department of Infectious Diseases and Clinical Microbiology, Faculty of Medicine, Kocaeli University, Kocaeli, Turkey; Department of Infectious Diseases and Clinical Microbiology, Ondokuz Mayis University, Samsun, Turkey; Department of Infectious Diseases and Clinical Microbiology, Istanbul Medeniyet University, Istanbul, Turkey

**Keywords:** *Staphylococcus aureus*, Bacteremia, Risk factors, Mortality, Sepsis

## Abstract

**Background:**

*Staphylococcus aureus* is one of the causes of both community and healthcare-associated bacteremia. The attributable mortality of *S. aureus* bacteremia (SAB) is still higher and predictors for mortality and clinical outcomes of this condition are need to be clarified. In this prospective observational study, we aimed to examine the predictive factors for mortality in patients with SAB in eight Turkish tertiary care hospitals.

**Methods:**

Adult patients with signs and symptoms of bacteremia with positive blood cultures for *S. aureus* were included. All data for episodes of SAB including demographics, clinical and laboratory findings, antibiotics, and outcome were recorded for a 3-year (2010–2012) period. Cox proportional hazard model with forward selection was used to assess the independent effect of risk factors on mortality. A 28-day mortality was the dependent variable in the Cox regression analysis.

**Results:**

A total of 255 episodes of SAB were enrolled. The median age of the patients was 59 years. Fifty-five percent of the episodes were considered as primary SAB and vascular catheter was the source of 42.1 %. Healthcare associated SAB was defined in 55.7 %. Blood cultures yielded methicillin-resistant *S. aureus* (MRSA) as a cause of SAB in 39.2 %. Initial empirical therapy was inappropriate in 28.2 %. Although overall mortality was observed in 52 (20.4 %), 28-day mortality rate was 15.3 %. Both the numbers of initial inappropriate empirical antibiotic treatment and the median hours to start an appropriate antibiotic between the cases of fatal outcome and survivors after fever onset were found to be similar (12/39 vs 60/216 and 6 vs 12 h, respectively; p > 0.05). High Charlson comorbidity index (CCI) score (p = 0.002), MRSA (p = 0.017), intensive care unit (ICU) admission (p < 0.001) and prior exposure to antibiotics (p = 0.002) all were significantly associated with mortality. The Cox analysis defined age [Hazard Ratio (HR) 1.03; p = 0.023], ICU admission (HR 6.9; p = 0.002), and high CCI score (HR 1.32; p = 0.002) as the independent predictive factors mortality.

**Conclusions:**

The results of this prospective study showed that age, ICU stay and high CCI score of a patient were the independent predictors of mortality and MRSA was also significantly associated with mortality in SAB.

## Background

*Staphylococcus aureus* is a leading cause of both community and healthcare -associated bacteremia and competes with *Escherichia coli* for the leading cause of community-acquired bacteremia. *S. aureus* bacteremia (SAB) can seed to virtually any body site and result in complications that may further result in severe disease, significant morbidity and death [1].

SAB places a substantial burden on health care systems with its high mortality rates of around 20–30 % and morbidity rates [2, 3]. This burden is increased by life-threatening complications, including infective endocarditis (IE) and other serious metastatic infections complications that more frequently require intensive care unit (ICU) admission and carry poor prognosis because of the anatomic site or the difficulty in reaching a timely diagnosis [4–6]. It is therefore imperative to know which patients are prone to SAB and its complications, variation of laboratory findings during SAB and its predictors of mortality.

Data regarding the characteristics of SAB in Turkey is insufficient and most of them are retrospective character [7–9]. For this reason, this prospective multicenter observational study aimed to identify predictors of mortality in patients with SAB in Turkish hospitals. The most common sources of SAB, its complications, and treatment responses to various antistaphylococcal agents were also investigated.

## Methods

### Study design and patients

This prospective, multicenter cohort study involved a 3-year period from January 2010 to December 2012. A thorough patient data collection form was sent and data were collected prospectively from eight participant centers in Turkey. All consecutive patients (15 years and over) with signs and symptoms of bacteremia with positive blood cultures for *S. aureus* were included in this study. Only the first clinically significant episode of infection with SAB for each patient was included in the analysis. Patients who had SAB as part of a polymicrobial bloodstream infection were excluded. Clinical Research Ethics Committee of Medipol University (Istanbul, Turkey) approved this study.

### Clinical and laboratory analyses and follow-up period

Patients with SAB were identified from microbiology laboratory records by daily visits. Patients were followed in the participant centers until discharge or death. Baseline characteristics, treatment regimens, outcome and other factors were recorded prospectively.

Usually two or more sets of blood cultures were obtained simultaneously from different sites and repeated when needed. Blood specimens were cultured on vials of automatic systems for a 7-day period in different centers, mainly by the BACTEC 9240 system (Becton–Dickinson, Maryland, USA). All *S. aureus* isolates were identified by standard laboratory methods at the clinical microbiology laboratory of participating centers. Antimicrobial susceptibilities of the isolates including oxacillin and vancomycin were determined by using disk diffusion test or automated systems according to the criteria of the Clinical and Laboratory Standards Institute (CLSI) [10]. During the study period all *S. aureus* isolates were submitted to the microbiology laboratory of Istanbul Medipol University for further verification. The identification and antimicrobial susceptibilities of isolates were performed by using Vitek 2 (bioMérieux, Marcy L’E`toile, France) bacterial identification and antimicrobial susceptibility testing (AST) system. Other specimens including pus, wound swabs, sputum, tracheal aspirates, bronchoalveolar lavage fluid, synovial fluid and central venous catheter (CVC) tips were also cultured to determine the source of SAB when necessary.

In order to determine the involvement of the cardiovascular system, transthoracic echocardiography (TTE) was performed for all patients when blood cultures were positive for *S. aureus*. Transesophageal echocardiography (TEE) was also performed for patients suspected of IE with negative TTE findings. Routine laboratory analyses and chest X-rays were also performed for all patients. The validated Charlson comorbidity index (CCI) score, which stratifies the associated diseases into an ordinal scale, was used to evaluate comorbidity in 28-day mortality among the patients [11]. Empirical antibiotic treatment was initiated following the diagnosis of infection and modified when needed.

### Definitions

#### Terms

Bacteremia was defined as the presence of ≥1 positive blood culture for *S. aureus* in a patient who had signs and symptoms consistent with an infection [12]. SAB was classified as community-acquired if *S. aureus* was isolated from blood cultures drawn within 48 h of admission of a patient with suggestive symptoms or signs of an infection after hospital admission, if the patient was not transferred from another hospital, and if the patient had any symptoms or signs suggestive of infection at admission. Otherwise, SAB was considered to have been health-care associated [13]. In the presence of a laboratory-confirmed bloodstream infection, primary bacteremia was considered if the organism cultured from blood is not related to an infection at another site. Otherwise, bacteremia was considered secondary. The diagnosis of Systemic Inflammatory Response Syndrome (SIRS) was made according to the criteria of the Surviving sepsis campaign and the presence of SIRS accompanied by infection was diagnosed as sepsis [14]. Prior admission history was defined as hospitalization within 90 days before the onset of SAB.

#### Foci of bloodstream infection

The primary foci of infection were determined using the following definitions. Catheter-related bloodstream infection (CR-BSI) was defined as SAB in any patient who has an intravascular device with ≥1 positive blood culture result obtained from the peripheral vein and no apparent source for SAB except the catheter. A microbiological proof of a catheter infection was warranted as either a positive result of a semiquantitative (>15 cfu per catheter segment) catheter culture whereby the same organism is isolated from a catheter segment and a peripheral blood culture, or a differential time to positivity (>2 h between catheter vs peripheral blood). Soft tissue infection was considered the source of SAB in cases where patients (a) had a culture of *S. aureus* from a tissue or a drainage specimen from the affected site and (b) had signs of infection [13]. Surgical site infection was defined according to the definitions of the Centers for Disease Control and Prevention (CDC) [15].

#### Antibiotic treatment and outcome

Empirical antibiotics were prescribed by the primary care physician. Appropriate antibiotic treatment was considered if the empirical therapy provided after the onset of bacteremia symptoms included at least one antibiotic to which the isolate was susceptible (a glycopeptide or a lipopeptide for methicillin resistant *S. aureus* (MRSA); beta-lactam/beta-lactamase inhibitor (BL/BLI) combination or cefazolin for methicillin susceptible *S. aureus* (MSSA).

De-escalation of antibiotic treatment was defined as a switch to a narrower spectrum agent and it was considered when an antibiotic with activity against MRSA was replaced by an antibiotic with activity against MSSA. Escalation of antibiotic treatment was defined as either the addition of a new antibiotic or a switch on an agent with MRSA activity.

The primary endpoint of this study was 28-day all-cause in-hospital mortality. Bacterial eradication was defined as the absence of *S. aureus* in repeated blood cultures during therapy and no evidence of recurrent SAB during a follow-up of 28 days, bacterial recurrence was defined as clinical resolution of signs and symptoms of infection during therapy but recurrent SAB during follow-up period. Treatment failure was defined as continuation of signs and symptoms of infection despite therapy or death.

### Statistical analysis

Statistical analysis was performed with Stata 13.1 (Stata Corp. LP, USA). Descriptive statistics such as means, standard deviation, medians, interquartile ranges (IQRs), frequencies, and percentages were collected. Comparisons between the continuous variables were performed using independent-samples *t* tests or Mann–Whitney *U* tests as appropriate, while comparisons between categorical variables were performed by Pearson’s Chi squared or Fisher’s exact tests. Cox proportional hazard model was used to assess the independent risk factors on mortality. Variables that have a significance value equal or less than 0.1 in the univariate analysis or variables that have been reported significant in the literature were included in the Cox proportional hazard analysis. Proportionality was controlled. Relative risks (RRs) and hazard ratios (HRs) with corresponding 95 % confidence intervals (95 % CIs) were calculated in order to describe the strength and direction of the association. Difference according to ICU stay was assessed by Kaplan–Meier survival curves and log-rank test. All statistical tests were two-tailed and a p value of <0.05 was accepted as statistically significant.

## Results

### Epidemiologic and descriptive data

During the study period 266 episodes of SAB were confirmed. Eleven were excluded from the analysis due to SAB as part of a polymicrobial bloodstream infection. Therefore, 255 patients (159 male, 96 female) with clinically significant of SAB episodes were analyzed. The median (IQR) age was 59 (45, 70) years. During the study period, death occurred in 52 (20.4 %) out of 255 cases. However a 28-day of mortality rate was 15.3 % (39 out of 255) for this study. Main descriptive characteristics of the 255 patients are given in Table 1. The number of patients over 65 years was 93 (36.5 %). The commonest comorbid condition was diabetes mellitus (24.7 %) among the cases. Fever (>38 °C) was present in 230 (90 %), tachycardia (>100 beats/min) in 191 (74.9 %), tachypnea (>20 breaths/min) in 147 (57.7 %), hypotension (arterial tension <90 mmHg) in 91 (35.7 %), and altered consciousness in 41 (16.1 %) patients at the time of blood culture collection. The most common coexistent factors for SAB among the patients were the presence of peripheral venous catheter (64.7 %) and CVC (51 %). Further, 107 (42 %) patients stayed in the ICU and 101 (39.6 %) patients had prior antibiotic use a month before of a blood culture was drawn. Routine laboratory analyses at the time of diagnosis revealed leukocytosis (>10,000/mm^3^) in 180 (70.6 %) and thrombocytopenia (<150,000/mm^3^) in 93 (36.5 %) out of 255 patients, respectively.

**Table 1:** Descriptive characteristics of the cohort (N = 255)

Variable	Number	Percent
Age >65 years	93	36.5
Comorbid diseases		
Diabetes mellitus	63	24.7
Chronic renal failure	56	22.0
Previous surgery	54	21.2
Malignant diseases	53	20.8
Skin disorder	51	20.0
Immunosuppressive therapy in last 3 months^a^	32	12.6
Burns	28	11.0
Chronic obstructive pulmonary disease	11	4.1
Congestive heart failure	11	4.1
Cerebrovascular event	10	3.9
Others^b^	50	19.6
Clinical findings		
Fever (≥38 °C)	230	910
Tachycardia (>100 beats/min)	191	74.9
Tachypnea (>20 breaths/min)	147	57.7
Hypotension (arterial tension <90 mmHg)	91	35.7
Altered consciousness^c^	41	16.1
Coexistent factors		
Peripheral venous catheter	165	64.7
Central venous catheter	130	51.0
Intensive care unit admission	107	42.0
Prior antibiotic use^d^	101	39.6
Mechanical ventilation	80	31.4
Prior hospitalization	78	30.6
Endotracheal tube	73	28.63
Tracheostomy	41	16.1
Prior cardio-pulmonary resuscitation	28	11.0
Laboratory findings		
Leukocytosis (WBC count >10,000/mm^3^)	180	70.6
Thrombocytopenia (platelet <150,000/mm^3^)	93	36.5
Leukopenia (WBC <4000/mm^3^)	26	10.2

*WBC* white blood cells

^a^Cancer chemotherapy, steroid treatment and others

^b^Cirrhosis, 9; Chronic alcoholism, 9; Connective tissue disorder, 6; Resident of elderly care center, 6; Alzheimer’s disease, 6; intravenous drug user, 2; Renal transplantation, 1

^c^Drowsiness and coma diagnosed by a physician

^d^A month prior to detection of *S. aureus* bacteremia

### Clinical characteristics

Of the 255 patients, 100 (39.2 %) had MRSA bacteremia and 155 (60.8 %) had MSSA bacteremia. One hundred forty-one patients (55 %) were considered as primary SAB. Of all remaining 114 (45 %) patients with secondary SAB; 48 (42.1 %) patients were diagnosed as CR-BSI, 30 (26.3 %) patients were diagnosed as skin and soft tissue infection (SSTI), 18 (15.8 %) were diagnosed as IE, and the remaining 18 (15.8 %) were diagnosed as surgical site infections (SSIs) (Data not shown).

### Microbiological and routine laboratory characteristics

A total of 515 (84.8 %) out of 607 blood cultures taken from the 255 patients grew *S. aureus*. The median (IQR) culture sets taken from the patients was 2 (2, 3) and the median (IQR) time to positivity of blood cultures of 170 patients with SAB was 24 (18, 48) hours. The average of 55.7 % of the episodes was healthcare-associated SAB. Ninety-five (95 %) of 100 MRSA and 47 (30.3 %) of 155 MSSA isolates were considered as health-care associated. Five percent of MRSA and 69.7 % of MSSA isolates were also considered as community-acquired. Routine laboratory parameters for patients who died and survived are compared in Table 2. The median highest white blood cells (WBC) count (p = 0.026), the mean lowest platelet counts (p < 0.0001) and the mean blood albumin levels (p = 0.0001) were significantly associated with death. The groups did not differ in terms of lowest WBC counts, highest serum creatinine, liver enzymes, alkaline phosphatase and C-reactive protein (CRP) levels (p > 0.05 for all comparisons).

**Table 2:** Comparison of routine laboratory parameters for fatal and survivor (28-day mortality) patients with *Staphylococcus aureus* bacteremia measured at the time of blood culture collection

Variable	Fatal (N = 39)	Survivor (N = 216)	p value	Total
Median (IQR) highest WBC (×10^9^/L)	18.8 (11.7, 23.4)	13.7 (10, 17.9)	*0.026*	14 (10, 18.8)
Median (IQR) lowest WBC (×10^9^/L)	8.8 (4.1, 13.3)	7.2 (5.4, 9.5)	0.287	7.3 (5.4, 9.6)
Mean ± SD (range) lowest platelet (×10^9^/L)	110 ± 94 (17–461)	196 ± 102 (5–545)	<*0.0001*	181 ± 106 (5–545)
Mean highest ± SD (range) BUN (mg/dL)	90 ± 72.2 (23–332)	65 ± 59.2 (7–383)	*0.03*	69.1 ± 61.8 (7–383)
Mean highest ± SD (range) creatinine (mg/dL)	2.1 ± 1.9 (0.3–6.7)	2.1 ± 2.5 (0.1–13.5)	0.929	2.1 ± 2.4 (0.1–13.5)
Mean highest ± SD (range) ALT (IU/L)	84.0 ± 246.6 (3–1497)	60.6 ± 106.5 (5–1160)	0.350	64.3 ± 137.6 (3–1497)
Mean highest ± SD (range) AST (IU/L)	177 ± 724 (6–4391)	69.2 ± 152.7 (6–1157)	0.062	86.1 ± 318.3 (6–4391)
Mean highest ± SD (range) GGT (IU/L)	106 ± 106.2 (6–422)	92.7 ± 111.3 (6–757)	0.613	94.5 ± 110.4 (6–757)
Mean highest ALP ± SD (range) (U/L)	177.6 ± 189 (46–686)	139.4 ± 119.8 (28–923)	0.221	144.2 ± 130.2 (28–923)
Mean lowest ± SD (range) albumin (g/dL)	2.5 ± 0.7 (1.1–4.6)	2.9 ± 0.6 (1.4–4.8)	*0.0001*	2.8 ± 0.7 (1.1–4.8)
Median (IQR) highest CRP (mg/L)	191.5 (131, 215)	171 (89, 218)	0.225	174 (101, 218)

Significant p values are presented as italics (p < 0.05)

*IQR* interquartile range, *SD* standard deviation, *WBC* white blood cells, *CRP* C-reactive protein, *ALT* alanine aminotransferase, *AST* aspartate aminotransferase, *ALP* alkaline phosphatase, *GGT* gamma-glutamyl transpeptidase, *BUN* blood urea nitrogen

### Characteristics of fatal and survivor patients

The results of evaluated demographical and clinical data in a univariate analysis are given in Table 3. ICU admission (p < 0.001), MRSA as a cause of SAB (p = 0.017) and history of exposure to antibiotics within last 30 days (p = 0.002) were all significantly associated with death. Although the number of patients having a minimum of one comorbid condition between the groups were the same (p = 0.102), the CCI scores were found to be different and fatal cases significantly had higher CCI score (p = 0.013). Both fatal and survivor groups were found to be very similar in terms of age and gender and initial inappropriate empirical antibiotic treatment (p > 0.05 for all comparisons). The median hours to start an appropriate antibiotic after fever onset between the fatal and survivor groups was also found to be not significant (6 vs 12 h; p = 0.437). Furthermore, the median (IQR) time to appropriate antibiotic after a positive culture for *S. aureus* was 16 (1, 96) hours in fatal group (n = 29) and it was 24 (1, 144) hours in survivor group (n = 131; p = 0.535) (Data not shown).

**Table 3:** Comparison of variables between survivor and patients with fatal outcome (28-day mortality) (N = 255)

Variable	Fatal	Survivor	p value
	(n = 39)	(n = 216)	
Median (IQR) age (years)	62 (53, 70)	59 (44, 70)	0.184
Gender, *n* (%)			0.552
Man	26 (66.7)	133 (61.6)	
Woman	13 (33.3)	83 (38.4)	
Median (IQR) time to appropriate antibiotic after fever onset (hours)	6 (2, 30)	12 (2, 27)	0.437
Inappropriate empirical antibiotic	12 (30.8)	60 (27.8)	0.703
Comorbid condition, *n* (%)^a^	34 (87.2)	160 (74.1)	0.102
Charlson comorbidity index score, *n* (%)			*0.013*
Low (0–1)	9 (23.1)	100 (46.3)	
Medium (2–4)	19 (48.7)	85 (39.4)	
High (≥5)	11 (28.2)	31 (14.3)	
Methicillin-resistant *S. aureus, n* (%)	22 (56.4)	78 (36.1)	*0.017*
Intensive care unit stay, *n* (%)	28 (71.8)	79 (36.6)	<*0.001*
Exposure to antibiotics, *n* (%)^b^	24 (61.5)	77 (35.6)	*0.002*

*IQR* interquartile range

Significant p values are presented as italics (p < 0.05)

^a^At least one comorbid condition per patient

^b^Within the last month at least for 3 days

### Complications of bacteremia

SAB resulted in complications in 24 (9.4 %) patients. Nine (37.5 %) patients developed pneumonia, 4 (16.7 %) patients developed vertebral osteomyelitis, 1 (4.2 %) patient developed splenic abscess, and one patient (4.2 %) developed endophthalmitis. The remaining 9 (37.5 %) patients developed other metastatic suppurative complications. Four patients showed a recurrence of bacteremia following cessation of treatment.

### Treatment and outcome

Following the assessment of the patients with SAB, 91 patients received glycopeptides or lipopeptides (vancomycin, teicoplanin, and daptomycin), 76 patients received BL/BLI combinations (ampicillin/sulbactam, cefoperazon/sulbactam, piperacillin/tazobactam, ticarcillin/clavulanate), 40 patients received BL antibiotics (cefazolin, imipenem or meropenem, ceftriaxone, cefuroxime, ceftazidime, cefepime, amoxicillin), 11 patients received intravenous linezolid and remaining 37 patients received various antibiotics as an empirical therapy. We were not able to show any differences regarding mortality between survivor and fatal groups based on the empirical antibiotics (p > 0.05 for all comparisons). Only the patients receiving empirical linezolid showed borderline statistical significance towards increased mortality (p = 0.05; RR = 2.4) (Table 4). Empirical antibiotic treatment was considered to be inappropriate in 72 (28.2 %) patients. In overall, empirical antibiotics were modified in 66 (25.9 %) patients. The spectrum of empirical treatment was deescalated in 3 (5.9 %) fatal and in 17 (8.4 %) survivors (p = 0.773) and escalated in 19 (36.5 %) fatal and in 27 (13.3 %) survivors (p < 0.0001) (Table 4). Bacterial eradication was achieved in 199 patients while empirical antibiotic treatment failed in 39 (100 %) fatal and in 11 (5.4 %) survivors (p < 0.0001). SAB recurred in 3 of the (7.7 %) fatal and 1 of the (0.5 %) survivors (p = 0.028) (Data not shown).

**Table 4:** Comparison of survivor and fatal patients with *Staphylococcus aureus* bacteremia in regards to empirical antibiotic treatments and modifications (60-day mortality) (N = 255)

Variable	Fatal (N = 52)	Survivor (N = 203)	Relative risk	[95 % CI]	p value
Empirical treatment options					
Peptide antibiotics	22 (42.3)	69 (33.9)	1.32	[0.81–2.15]	0.264
BL/BLI	10 (19.2)	66 (32.5)	0.56	[0.29–1.06]	0.062
BL	9 (17.3)	31 (15.3)	1.13	[0.59–2.12]	0.719
Linezolid	5 (9.6)	6 (3.0)	2.36	[1.18–4.74]	0.05
Others^a^	6 (11.5)	31 (15.3)	1.1	[0.91–1.24]	0.495
Antibiotic modifications					
De-escalated	3 (5.9)	17 (8.4)	0.67	[0.26–2.19]	0.773
Escalated	19 (36.5)	27 (13.3)	2.61	[1.64–4.17]	<*0.0001*
Unchanged	30 (57.6)	159 (78.3)	0.48	[0.29–0.76]	*0.0024*

Peptide antibiotics: vancomycin, teicoplanin, and daptomycin

*BL/BLI* beta lactam/beta lactamase inhibitors, *BL* beta lactam antibiotics, *CI* confidence interval

Data are presented as *n* (%). Significant p values are presented as italics (p < 0.05)

^a^Fluoroquinolone (ciprofloxacin, levofloxacin, moxifloxacin), 16; Tigecycline, 10; Colistin, 10

Effects of empirical antibiotic treatment with respect to oxacillin susceptibility of *S. aureus* on 28-day mortality of our cohort are summarized in Table 5. Although inappropriate empirical therapy was associated with mortality in one-thirds of the patients in both MRSA (7/22) and MSSA (5/17) bacteremia groups, this effect was not statistically significant (p > 0.05). Gentamicin was added to treatment for various reasons in 15 patients. The overall 14-, 28- and 60-day mortality rates were 11 % (28/255), 15.3 % (39/255), and 20.4 % (52/255) respectively. However 80 (31.4 %) patients with severe sepsis or septic shock had a 28-day mortality rate of 32.5 % (26 patients). Inappropriate empirical antibiotic treatment in 23 (28.8 %) of 80 patients was associated with 8 (34.8 %) deaths. Further, the median (IQR) time to appropriate antibiotic after a positive culture for *S. aureus* was 12 (3, 24) hours in patients with severe sepsis or septic shock and it was 10 (2, 36) hours in patients without severe sepsis or septic shock (p = 0.73) (data not shown). Among all patients who died, death was definitely attributed to SAB in 6/52 (11.5 %) patients, probably attributed to SAB in 20/52 (38.5 %) patients and to other reasons including underlying diseases in 21/52 (40.3 %) patients. The exact cause of death was unidentified in the remaining 5/52 (9.6 %) patients.

**Table 5:** Appropriate empirical antibiotic use with respect to oxacillin susceptibility and outcome of *Staphylococcus aureus* bacteremia (28-day mortality)

Appropriate antibiotic	Oxacillin-resistant (N = 100)			Oxacillin-susceptible (N = 155)		
	Fatal	Survivor	p value	Fatal	Survivor	p value
Yes	15 (25.4)	44 (74.6)	0.322	12 (9.7)	112 (91.3)	0.336
No	7 (17.1)	34 (82.9)		5 (16.1)	26 (83.9)	

Data presented as *n* (%) of row

**Fig. 1:**
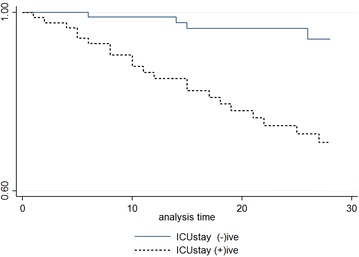
Kaplan–Meier survival estimates of patients regarding intensive care unit (ICU) stay

In a multivariable Cox model the independent predictors of 28-day all-cause of mortality were: age (HR 1.03; p = 0.023), ICU stay (HR 6.9; p = 0.002), and high CCI score (HR 1.32; p = 0.002) (Table 6). Kaplan–Meier survival estimates of patients with SAB regarding ICU stay is shown in Fig. 1.

**Table 6:** Cox proportional hazard model for 28-day mortality

Variable	Hazard ratio	[95 % CI]	p value
Age (per year increase)	1.03	[1.0–1.05]	*0.023*
Intensive care unit stay	6.9	[2.02–23.6]	*0.002*
Time to appropriate antibiotic after fever onset (hours)	1.0	[0.99–1.02]	0.833
Exposure to antibiotics^a^	1.98	[0.73–5.42]	0.181
Methicillin-resistant *S. aureus*	0.99	[0.33–3.0]	0.991
Charlson comorbidity index score	1.32	[1.1–1.58]	*0.002*

*CI* confidence interval, significant p values are presented as italic (p < 0.05)

^a^Within last 30 days at least for 3 days

## Discussion

*Staphylococcus aureus* bacteremia is a common and serious infection with significant morbidity and mortality, especially in ICU patients [16]. It is difficult to determine the exact incidence of SAB, since prospective population-based surveillance studies are infrequently performed. The annual incidence has been reported as low as 19.7/100,000 population in Canada and 26/100,000 population in Sweden [17–19], and as high as 50/100,000 population in the USA [20]. The difference could be due to infection control practices or variances in surveillance systems. Interestingly, the incidence of SAB has generally been reported to be higher for males than for females, while some studies have shown an increased mortality for females [20, 21]. Sixty-two percent of our cohort was men and the mortality of SAB in men and women were 16.4 % and 13.5 %, respectively. We have not detected any gender difference in regard to outcomes.

The SAB episodes due to MSSA is generally predominate, especially in countries with a low prevalence of MRSA [17, 22]. Likewise, 60.8 % of our patients turned out MSSA when consecutive 255 patients with SAB were collected. Mortality of SAB has been reported to be declining steadily throughout the 20th century, probably as a result of greater understanding of SAB management. Case-mortality associated with hospital-acquired and community-acquired SAB showed rate reductions of 43 and 23 %, respectively between 1981 and 2000 (p = 0.0001) [17]. A previous prospective study suggests 30-day all-cause mortality of 20.6 % which is significantly associated with older age, and MRSA infection as we found in the cohort [23]. A recent prospective study that included patients with both community and healthcare-associated SAB between 2007 and 2011 reported 30-day all-cause and infection-related mortalities of 25.8 and 11.2 %, respectively [24]. Our cohort showed 28-day all cause and infection-related mortalities of 15.3 and 10.2 %, respectively which may suggest that mortality of SAB may be further decreasing. However, prospective data from larger cohorts are required to resolve this issue. Age has been confirmed as a strong independent predictor of mortality in many studies as we found [23, 25, 26]. The mortality rate was found to be increased from 6 % in young individuals (<15 years old) to 57 % in adults older than 85 years of age by Lamagni et al. [25].

It is well known that presence of comorbidities can influence the patient outcome. The SAB mortality has been accepted to increase with the presence of one or multiple comorbidities including immunosuppression, cirrhosis, malignancy, and chronic renal failure [17, 27–29]. This study showed that the number of patients having a minimum of one comorbid condition between the fatal and survivor groups were the same. However CCI scoring for the assessment of the severity of illness indicator [11] revealed that cases with fatal outcome had significantly high CCI score suggesting these cases had more severe illness. However, other studies detected any difference in outcomes for patients with comorbidities [30–34].

Ninety-five percent of MRSA were isolated from healthcare-associated SABs, while 70 % of MSSA were isolated from community-acquired SABs in our cohort. According to current cohort studies, the setting of SAB onset, whether health-care associated or community-acquired, does not seem to have an effect on patient outcomes [12, 34, 35]. Likewise, we were not able to show any difference in mortality rates based on the setting of SAB onset. However, advent of community acquired (CA)-MRSA may change the equation, which remains to be shown in future studies. Currently CA-MRSA isolates are very rarely reported in Turkey [36].

Patients with SAB developing sepsis or septic shock have mortality rates of ranging between 38 and 86 %, and are strongly associated with worse outcomes [32, 37]. Eighty (31.4 %) patients developed severe sepsis or septic shock in our cohort and 28-day mortality was 32.5 % for such cases. A previous retrospective Turkish study from a single center reported 41.7 % of mortality rate for patients with septic shock [38].

The impact of methicillin resistance on mortality of SAB has been examined by a number of studies. The results of the majority of these studies are conflicting. A well-designed meta-analysis by Cosgrove et al. [39] showed that mortality rates for MRSA bacteremia were significantly higher than that of MSSA bacteremia (OR, 1.93; 95 % CI, 1.54–2.42; p < 0.001). Methicillin resistance was found to be an independent risk factor of mortality in our cohort. This finding could be explained by differences in empirical prescribing [40] or poor vancomycin efficacy [41]. However, a recent study by Wolkewitz et al. [42] showed that there is only a small and statistically insignificant difference between mortality rates of MRSA and MSSA bacteremia when length of hospital stay was adjusted. Similarly a large prospective study by Melzer et al. [43] also revealed that MRSA bacteremia was not associated with increased mortality after adjustments for various host confounders including comorbidities, age, and severity of illness.

Heteroresistant vancomycin-intermediate S. aureus (hVISA) should also be considered as a factor for SAB mortality. Although mortality rate of SAB due to hVISA has been reported as high as 75 % in earlier studies [44], overall, hVISA bacteremia is not associated with an increased mortality rate [45]. One of the limitations in our study was that antimicrobial susceptibility testing for all *S. aureus* isolates was performed by disk diffusion or commercial automated systems, which tend to undercall resistance [46]. Therefore we are not able to discuss any effect of hVISA on mortality of our patients.

Complications of SAB are common, occurring at rates that range from 11 to 53 % [6, 47]. A recent study evaluating predictive factors associated with the development of metastatic infection following SAB revealed a delay in appropriate antimicrobial treatment of >48 h, persistent fever for >72 h after starting antibiotic treatment and lowest CRP levels of >3 mg/dL as significant in multivariate analysis [48]. Some complications more frequently require intensive care admission and carry poor prognosis because of the anatomic site or the difficulty in reaching a timely diagnosis.

*Staphylococcus aureus* is one of the only causes of IE in structurally normal heart valves [49], and it is the leading cause of IE in several countries [5]. A large-scale prospective, multicenter, observational study showed that 13 % of patients with SAB had IE [50]. We found a similar incidence of 15.8 % in our study. Patients with hospital-acquired SAB have a lower incidence of IE than those with community-acquired SAB (2–17 %) as expected [50].

For the management of SAB, no antimicrobial agent was shown to result in better patient outcomes and the initial therapy was inappropriate in 28.2 % of 255 SAB episodes in our cohort. This was an unexpected observation because inappropriate antibiotic treatment was identified as an independent predictive factor for mortality, as in previous studies [32, 33]. A previous study performed in 819 MRSA bacteremia episodes showed that inappropriate antibiotic had no effect on mortality [30]. Early administration of broad-spectrum antimicrobials has always been stressed and advocated in surviving sepsis campaigns [51], however, evidence for this recommendation has been mainly provided in patients with septic shock [52–55].

In addition to inappropriate initial empirical antibiotic, our study did not show delayed time to appropriate antibiotic after fever onset as a predictor of mortality. This finding could be explained by an initial treatment selection bias. However, there are other studies that are unable to detect a mortality difference regarding timing of appropriate empirical therapy [32, 37]. When Lodise et al. looked at as to which patient subgroups would actually benefit greatest from timing of empirical antibiotics, they found that patients with severe disease benefitted most [56]. Only a minority of the patients in our cohort had severe sepsis and the remaining majority of patients might have neutralized the impact of delayed appropriate antimicrobial treatment in that group. About 80 % of patients with SAB do not suffer from severe sepsis or septic shock [57]. Patients with SAB caused by MRSA may be at increased mortality risk of being treated inadequately when MRSA is not covered in empiric antimicrobial regimens. However, community acquired MRSA is very rare in Turkey and the incidence of *S. aureus* infections is rapidly declining in Turkish ICUs [58]. Due to all the reasons stated above, our study may not have shown significant differences in outcome between patients with early and delayed appropriate antimicrobial treatments.

Intensive care unit stay was one of the independent risk factors of mortality in our study. Either transit to an ICU [59] or the acquisition of SAB in the ICU [12] has been found to be independent predictors of mortality, probably since they were surrogate markers for infection severity.

In conclusion, the data from this prospective study performed in Turkish patients demonstrated several prognostic factors for SAB mortality including age, ICU stay and high CCI scores. Furthermore, methicillin resistance may be a risk factor for mortality of patients with SAB. In contrast, inappropriate initial empirical antibiotics and delays of starting the appropriate antibiotics within a few hours may not have any impact on mortality. In order to proper management of SAB, clinicians should be aware of patients with one or some of the above mentioned predictors.
